# Casein Oligochitosan-Glycation by Transglutaminase Enhances the Anti-Inflammatory Potential of Casein Hydrolysates to the Lipopolysaccharide-Stimulated IEC-6 Cells

**DOI:** 10.3390/nu14030686

**Published:** 2022-02-06

**Authors:** Na Chen, Li Wang, Qiang Zhang, Xin-Huai Zhao, Jia Shi

**Affiliations:** 1 Key Laboratory of Dairy Science, Ministry of Education, Northeast Agricultural University, Harbin 150030, China; chenna108@163.com; 2 School of Biology and Food Engineering, Guangdong University of Petrochemical Technology, Maoming 525000, China; wangli7742@gdupt.edu.cn (L.W.); zhangqiang@gdupt.edu.cn (Q.Z.); 3 Research Centre of Food Nutrition and Human Healthcare, Guangdong University of Petrochemical Technology, Maoming 525000, China; 4 Maoming Branch, Guangdong Laboratory for Lingnan Modern Agriculture, Guangdong University of Petrochemical Technology, Maoming 525000, China

**Keywords:** casein, oligochitosan, glycation, IEC-6 cells, lipopolysaccharide, anti-inflammatory effect

## Abstract

In this study, milk protein casein was glycated by oligochitosan through the catalysis of transglutaminase (TGase) and then hydrolyzed by trypsin. The obtained glycated casein hydrolysates (GCNH) were assessed for their anti-inflammatory activities, using the lipopolysaccharide (LPS)-stimulated rat intestinal epithelial cells (IEC-6) as cell models and the casein hydrolysates (CNH) without TGase catalysis as controls. The results showed that GCNH had oligochitosan incorporation and thus possessed a glucosamine content of 5.74 g/kg protein. In general, GCNH at dose levels of 25–100 μg/mL could elevate IEC-6 cell growth, and at dose levels of 25–50 μg/mL, they were also able to alleviate the LPS-induced cytotoxicity by increasing cell viability efficiently. Although LPS caused clear inflammation in the LPS-stimulated cells, GCNH were capable of reducing the secretion of three pro-inflammatory mediators including interleukin-1β (IL-1β), IL-6, and tumor necrosis factor-α, or promoting the secretion of two anti-inflammatory mediators like IL-10 and transforming growth factor-β, demonstrating their anti-inflammatory activities to the stimulated cells. Moreover, GCNH also could down-regulate the expression of three inflammation-related proteins including TLR4, p-p38, and p-p65 in the stimulated cells, and thus possessed a capacity to suppress the phosphorylation of p38 and p65 proteins as well as to inactivate the NF-κB and MAPK signaling pathways. Additionally, a higher GCNH dose level consistently led to higher anti-inflammatory effect in the cells, while GCNH were always more potent than CNH at performing anti-inflammatory function targets. It is thus suggested that the TGase-catalyzed casein oligochitosan-glycation could enhance the anti-inflammatory activities of casein hydrolysates efficiently. TGase-catalyzed protein glycation thus might enhance the healthcare function of protein ingredients in the body.

## 1. Introduction

Proteins as one main component of dietary foodstuffs are vital to the body, because they can provide the essential nutrients for life growth. In recent years, several protein modifications have been used to treat food proteins, aiming to improve protein functionalities or enlarge their application fields. In general, glycation, phosphorylation, and acetylation are the three main pathways, during which the saccharide, phosphate, and acetyl groups are covalently conjugated into amino acid residues of the protein targets, respectively. Subsequently, the modified proteins have changed configuration and more importantly functional properties. It was revealed that glycation, phosphorylation, and acetylation of proteins could improve these physicochemical properties of proteins like interfacial properties, solubility, surface hydrophobicity, and emulsification [1,2,3]. In addition, chemical modifications of proteins can also impact their digestion or bioactivities. For example, the performed glycation, phosphorylation, and acetylation of bovine α-lactalbumin caused an attenuation of its allergic response [4], while the used phosphorylation of soy protein isolate (SPI) led to reduced digestion in the intestine [5]. Moreover, it was reported that the Maillard-type protein glycation could cause the loss of the essential amino acids (e.g., lysine and arginine) in proteins [6], and resulted in protein digestion in the gastrointestinal tract [7]. Thus, the transglutaminase (TGase)-mediated protein glycation using the saccharides with amino groups was explored in the previous studies [1,8,9,10,11]. The possible effect of this TGase-catalyzed glycation on the biological functions of food proteins is also concerned by the researchers. The results showed that this enzymatic protein glycation could promote the anti-oxidant capacity of corn gluten meal [2]. The results of our group also showed that TGase-mediated casein oligochitosan-glycation could endow the casein hydrolysates with better barrier protection, higher growth proliferation and differentiation in the rat intestinal epithelial cells (IEC-6 cells) [12,13]. However, whether the TGase-induced protein glycation has a positive or negative impact on the anti-inflammatory function of the target proteins or protein hydrolysates is still insufficiently investigated so far. Such a study using the TGase-mediated case in oligochitosan-glycation thus deserves our consideration.

Inflammation is a series of physiological responses that are stimulated by the immune system when the body is stimulated by the harmful substances. Infection and tissue damage are the typical triggers of inflammation. Lipopolysaccharide (LPS) is a major component in the cell wall of the Gram-negative bacteria, can be recognized by cells, and thus induces cells to produce pro-inflammatory cytokines, which finally damage cells [14]. LPS is thus considered to be an effective activator of the inflammatory response [15]. When LPS induces an inflammation in macrophages, the cells will release a large number of inflammatory markers such as cytokines, NO, prostaglandin E2 (PGE2), cyclooxygenase (COX-2), and others. The related inflammatory signaling pathways like NF-κB and MAPK are thus activated. It is recognized that inflammation is associated with many chronic diseases such as cancer, obesity, and gastrointestinal diseases. Fortunately, several food components, such as proteins [16], polysaccharides [17], and polyphenols, [18] are proven to have an ability to antagonize the induced inflammation. It was found that mung bean protein hydrolysates [19], sturgeon protein-derived peptides [20], and sea buckthorn flavonoids [21] were able to regulate the expression of inflammatory markers in the LPS-stimulated RAW 264.7 macrophages and then activate the relevant signaling pathways to exert an anti-inflammatory effect. In addition, the polysaccharides from strawberry and mulberry juice showed anti-inflammatory effects on the LPS-stimulated spleen cells by inhibiting the secretion of inflammatory cytokines but enhancing the secretion of anti-inflammatory cytokines [22], while β-carotene exerted an anti-inflammatory effect on the LPS-stimulated IEC-6 cells via modulating autophagy and regulating signaling pathways [23]. Moreover, intestinal epithelial cells (such as IEC-6) have recently been used as cell models to investigate the intestinal inflammation of bovine lactoferrin [24] and echinacoside [25]. Thus, this cell model might also be suitable to investigate the anti-inflammatory effect of the oligochitosan-glycated casein hydrolysates.

Cytokines are important in the development and maintenance of inflammation. Currently, tumor necrosis factor (TNF-α) and interleukins (like IL-1β, IL-6 and IL-10) are widely used to reflect the anti-inflammatory effects of food ingredients [26], because the LPS–induced cellular inflammation can increase the secretion of these inflammatory factors IL-6, IL-1β, and TNF-α [20,27] but decrease the secretion of anti-inflammatory factors IL-10 and TGF-β [25]. Additionally, the expression of nitric oxide synthase (iNOS) and COX-2 is also closely associated with inflammatory response because their overexpression will promote the secretion of NO and PGE2 and then trigger a cellular inflammatory response [28]. It is worth noting that these cytokines and non-cytokine mediators ultimately are regulated by the extracellular signaling pathways [29]. Typically, the nuclear factor kappa-B (NF-κB) and mitogen-activated protein kinase (MAPK) pathways are two common extracellular signaling pathways involved in cellular inflammation [30]. Subsequently, the anti-inflammatory effects of irisin and the protein hydrolysates of brewer’s spent grain were evidenced to be mediated by the TLR4-NF-κB/MAPK signaling pathway [31,32].

In this study, the glycated casein hydrolysates obtained from the oligochitosan-glycated casein were assessed for their anti-inflammatory potential in the LPS-stimulated IEC-6 cells, using the unglycated casein hydrolysates as a control. Several indices including cell viability, cytokine secretion, and the expression of critical proteins related to TLR4/p38 MAPK/NF-κB signaling pathway were measured, with a purpose to identify whether the performed TGase-mediated glycation had positive or negative influence on anti-inflammatory function of casein in the intestine.

## 2. Materials and Methods

### 2.1. Materials and Reagents

Casein (protein content of 911.2 g/kg, dry basis), Dulbecco’s modified Eagle’s medium (DMEM), 3-(4,5-dimethyl-2-thiazolyl)-2,5-diphenyltetrazolium bromide (MTT), and lipopolysaccharide (LPS, derived from *E. coli* O55:B5) were purchased from Sigma-Aldrich Chemical Co. (St. Louis, MO, USA). Oliogochitosan (75% deacetylation and polymerization degree of 2–20) was obtained from Zhejiang Golden Shell Biochemical Co. (Hangzhou, Zhejiang, China). Microbial transglutaminase (TGase) with enzyme activity of 147 units (U)/g was the product of Jiangsu Yiming Fine Chemical Industry Co., Ltd. (Taixing, Jiangsu, China), while a trypsin (EC 3.4.21.4) preparation (120 kU/g) was purchased from Beijing Auboxing Biotechnology Co. (Beijing, China). Trypsin-EDTA (ethylenediamine tetra-acetic acid) and bovine insulin were provided by Solarbio Technology Ltd. (Beijing, China), while fetal bovine serum (FBS) was bought from Wisent Inc. (Montreal, QC, Canada).

Five enzyme-linked immunosorbent assay (ELISA) kits [rat tumor necrosis factor-α (TNF-α), transforming growth factor-β (TGF-β), and three interleukins (IL-1β, IL-6, and IL-10)] were bought from Nanjing Jiancheng Institute of Biological Engineering (Nanjing, China), while the protein assay kit (BCA) and radio immunoprecipitation assay (RIPA) lysis buffer were provided by Beyotime Biotechnology Institute (Shanghai, China). The primary antibodies of p-p65 (phospho-NF-κB p65, Bioss bs-0982R), TLR4 (Bioss bs-20594R) and β-actin (Bioss bs-0061R) were provided by Biosynthesis Biotechnology Inc. (Beijing, China), while phosphorylated p38 MAPK (#4511) and the goat anti-rabbit HRP secondary antibody (#7074) were obtained from Cell Signaling Technology (Danvers, MA, USA).

### 2.2. Sample Preparation

The oligochitosan-glycated casein was prepared as previously described [33]. In brief, casein was dispersed in water at pH 7.5 to reach 80 g/L. Oligochitosan was added into casein solution to reach the molar ratio of acyl donor to oligochitosan acceptor 1:3 and final protein content of 50 g/L. TGase (10 U/g protein) was added to perform the reactions at 37 °C for 3 h. The mixture was then heated at 85 °C for 5 min, cooled rapidly, and precipitated by adding 0.1 mol/L HCl solution to pH 4.5. The obtained precipitate (oligochitosan-glycated casein) was washed twice with water at pH 4.5, neutralized to pH 7.0, and then freeze-dried. Casein was also mixed with oligochitosan but without TGase addition, and then treated similarly to obtain control casein.

The control casein and glycated casein (50 g/L) were dispersed in water at pH 7.0, and then hydrolyzed at 37 °C for 4 h with trypsin addition of 7 kU/g protein. After the hydrolysis, the solutions were heated at 100 °C for 5 min, cooled rapidly to 20°C, neutralized to pH 7.0, and centrifuged at 5000× *g* for 20 min. The obtained supernatants were collected and lyophilized to obtain the respective casein hydrolysates (CNH) and oligochitosan-glycated casein hydrolysates (GCNH).

### 2.3. Assays of Protein and Glucosamine Contents

Protein content was determined by the Kjeldahl method with a conversion factor of 6.38 [34]. Glucosamine content was determined according to the Elson–Morgan method with minor modifications [35]. In detail, the sample of 0.2 g was hydrolyzed with 6 mol/L HCl of 2.5 mL at 100 °C for 4 h, cooled to 20 °C, neutralized to pH 7.0, and diluted with water into 25 mL. Acetylacetone solution (acetylacetone of 1 mL plus 0.5 mol/L NaHCO_3_ of 24 mL) of 1 mL was added to the hydrolysates of 5 mL, heated in a boiling water bath for 25 min, cooled rapidly, mixed with p-2-dimethylaminobenzaldehyde solution of 1 mL (0.8 g of p-dimethylaminobenzaldehyde in 12 mol/L HCl of 15 mL and aldehyde-free ethanol of 15 mL) and aldehyde-free ethanol of 3 mL, and then kept at 60 °C for 1 h. The absorbance value was measured at 525 nm by a UV spectrophotometer (UV-2600, Shimadzu, Kyoto, Japan). Glucosamine content (g/kg protein) was calculated using a standard curve generated from a series of standard glucosamine solutions.

### 2.4. Cell Line and Cell Culture

As recommended, IEC-6 cells provided by the American Type Culture Collection (Rockville, MD, USA) were cultured at 37 °C and 5% CO_2_ in the DMEM medium fortified with 4 mmol/L L-glutamine, 4.5 g/L glucose, 1.5 g/L NaHCO_3_, 1 mmol/L sodium pyruvate, 100 U/L bovine insulin and 10% fetal bovine serum. The medium was replaced twice a week, while the cells were fused to 80% for the later experiments.

### 2.5. Assays of Cell Viability

The classic MTT method was employed to determine the potential cytotoxicity of the two hydrolysates CNH and GCNH on IEC-6 cells. In brief, the cells (1 × 10^4^ cells/well) were seeded into 96-well plates for 24 h, and then serum-starved for 12 h. After this treatment, the cells were incubated with or without CNH and GCNH for 12–48 h, using the sample doses ranging from 25 to 100 μg/mL. After removal of the medium, MTT solution (0.5 mg/mL) of 20 μL was added to each well, while the cells were incubated for 4 h. After a careful aspiration of the medium, 100 μL of dimethyl sulfoxide (DMSO) was added to dissolve the generated formalin crystals, while the value of optical density (OD) was detected at 490 nm by a microplate reader (Bio-Rad Laboratories, Hercules, CA, USA). The control cells without sample treatment were set at 100% cell viability, as previously described [13].

### 2.6. Assay of LPS Cytotoxicity

Briefly, IEC-6 cells at a density of 1 × 10^4^ cells/well were seeded onto 96-well plates, incubated for 24 h, and serum-starved for 12 h. After that, the cells were incubated with or without CNH and GCNH at the doses of 25–50 μg/mL for 12 and 24 h and exposed to 10 μg/mL LPS for 24 h. The medium was discarded, while 0.5 mg/mL of MTT solution was added to each well with an incubation time of 4 h. DMSO of 100 μL was added to each well after a careful aspiration of the medium. The OD value was detected at 490 nm by the same microplate reader. The control cells without any treatment were set with 100% cell viability.

### 2.7. Assays of Cytokine Secretion

The secretion levels of three inflammatory mediators (IL-1β, IL-6, and TNF-α) and two anti-inflammatory mediators (IL-10 and TGF-β) were measured with the respective enzyme-linked immunosorbent assay (ELISA) kits. In brief, IEC-6 cells (1 × 10^5^ cells/mL, 2 mL/well) were loaded into 6-well plates for 24 h and incubated in serum-free medium for 12 h. After discarding the medium, CNH and GCNH at the doses of 25–50 μg/mL were added into each well to treat the cells for 12 and 24 h, followed by LPS exposure (10 μg/mL) of the cells for 24 h. Afterwards, the supernatants were collected by a centrifugation at 500× *g* for 20 min, while the cytokine levels were assessed using the instructions provided by the kit manufacturers.

### 2.8. Assays of Protein Expression

In brief, IEC-6 cells were plated into cell bottles for 24 h and incubated in serum-free medium for 12 h. After this treatment, the cells were incubated with or without CNH and GCNH at the doses of 25–50 μg/mL for 12 and 24 h, and then exposed to 10 μg/mL LPS for 24 h. After discarding the supernatants, the cells were washed three times with ice-PBS and lysed on ice for 30 min by adding lysis buffer containing protease inhibitor. The lysed cells were centrifuged at 12,000× *g* for 5 min at 4 °C to obtain total protein. A BCA protein analysis kit was used to assess the protein content of the samples. The equivalent samples with protein of 10 μg were subjected to 12% SDS-PAGE and subsequently transferred onto the PVDF membranes. After blocking with 5% skim milk in TBST (1 × TPBS containing 0.1% Tween-20) for 2 h at 37 °C, the membranes were kept overnight at 4 °C with primary antibody β-actin (1:1000 dilution), TLR4 (1:1000 dilution), p-p38 MAPK (1:1000 dilution) and p-p65 (1:500 dilution). Afterwards, the membranes were washed three times with TBST and incubated for 2 h at 37 °C with the peroxidase-conjugated secondary antibody (1:1000 dilution). The membranes were washed with TBST three times, while the immunolabeled proteins were detected with enhanced chemiluminescence reagents. Image J software (National Institutes of Health, Bethesda, MD, USA) was used for the quantitative analysis of protein bands.

### 2.9. Statistical Analysis

All experimental results from three independent experiments were expressed as the mean values ± standard deviations. Statistical analysis (one-way analysis of variance, ANOVA) and Duncan multi-interval test were performed using the IBM Statistical Products and Services Solutions (SPSS) 26.0 software. The *p* < 0.05 was considered statistically significant.

## 3. Results

### 3.1. Effect of the Two Hydrolysates on Cell Growth

**Figure 1:**
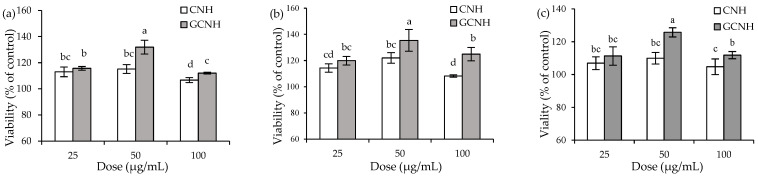
Viability values of the IEC-6 cells incubated with casein hydrolysates (CNH) and oligochitosan-glycated CNH (GCNH) at doses of 25–100 μg/mL for 12 (**a**), 24 (**b**), and 48 h (**c**), respectively. Different lowercase letters above the columns indicate that the mean values differ significantly (*p* < 0.05).

The results showed that the conjugated glucosamine amount of GCNH was 5.74 g/kg protein, suggesting that GCNH contained oligochitosan-glycated peptides. Thereby, it was possible that GCNH had different anti-inflammatory potentials than CNH in the target cells, because of the occurred oligochitosan glycation. When IEC-6 cells were incubated with CNH and GCNH at three doses of 25–100 μg/mL for three set time periods (12–48 h), all treated cells were detected with viability values larger than 100% (Figure 1), indicating that the two samples did not have a toxic effect on cells. To be more specific, when the cells were incubated with CNH and GCNH for 12 h, viability values were measured to be 106.7–115.2% and 112–131.9%, respectively (Figure 1a). A longer cell incubation time of 24 h would promote the viability values to 108.2–122% and 119.9–135.4% (Figure 1b), while another incubation time of 48 h caused viability values of 104.7–109.9% and 111.3–125.7%, respectively (Figure 1c). All data suggested that the two hydrolysates had a favorable but not suppressing effect on cell growth in all cases. Data comparison also demonstrated that GCNH had higher potential than CNH to promote cell growth, evidencing that the performed oligochitosan glycation caused an enhanced bioactivity for casein. At the same time, a cell treatment time of 48 h caused a lower viability values than that of 12 and 24 h, and more importantly, the doses of 25–50 μg/mL generally led to higher viability values than the dose of 100 μg/mL. Thus, two cell treatment times of 12 and 24 h together with two sample doses of 25 and 50 μg/mL were used in later evaluations to clarify and compare the anti-inflammatory activities of CNH and GCNH.

### 3.2. Effect of the Two Hydrolysates on the LPS-Induced Cellular Injury

**Figure 2:**
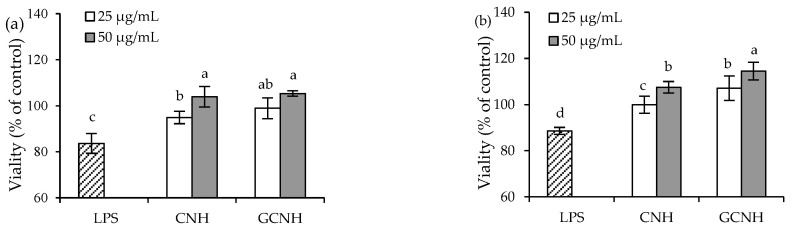
Viability values of the IEC-6 cells incubated with casein hydrolysates (CNH) and oligochitosan-glycated CNH (GCNH) at doses of 25–50 μg/mL for 12 (**a**) and 24 (**b**) followed by LPS treatment (10 μg/mL) of 24 h. Different lowercase letters above the columns indicate that the mean values differ significantly (*p* < 0.05).

Using the mentioned conditions to treat the cells or expose the cells with 10 μg/mL LPS for 24 h, the final results indicated that LPS caused cytotoxic effect on the cells, but the target hydrolysates could alleviate the LPS-caused adverse effect (Figure 2). In detail, the cells with LPS exposure but without hydrolysate treatment showed viability values of 83.6% (12 h) and 88.6% (24 h), reflecting the LPS-induced cell injury. When the cells were first treated with CNH and then exposed to LPS, they were measured with viability values of 94.9–103.9% (12 h) and 98.9–105.3% (24 h). At the same time, if the cells were pre-treated with GCNH, they were measured with viability values of 99.9–107.5% (12 h) and 107.1–114.5% (24 h). The data proved that the two hydrolysates alleviated the LPS-induced cell injury in all cases, and thus possessed protective effect on the cells. Generally, higher hydrolysate dose and longer treatment time consistently led to higher viability values. More importantly, GCNH were more active than CNH in protecting the LPS-induced cell injury, verifying that the performed oligochitosan glycation of casein caused higher bioactivity for GCNH.

### 3.3. Effect of Two Hydrolysates on the Secretion of Inflammatory Mediators

**Figure 3:**
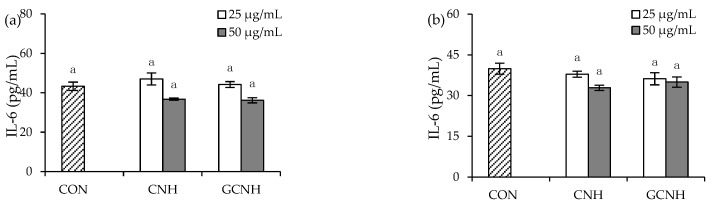
IL-6 secretion in the IEC-6 cells incubated with the casein hydrolysates (CNH) and oligochitosan-glycated CNH (GCNH) at doses of 25–50 μg/mL for 12 (**a**) and 24 h (**b**). The abbreviation “CON” denotes the control cells without sample treatment, while different lowercase letters above the columns indicate that the mean values differ significantly (*p* < 0.05).

**Figure 4:**
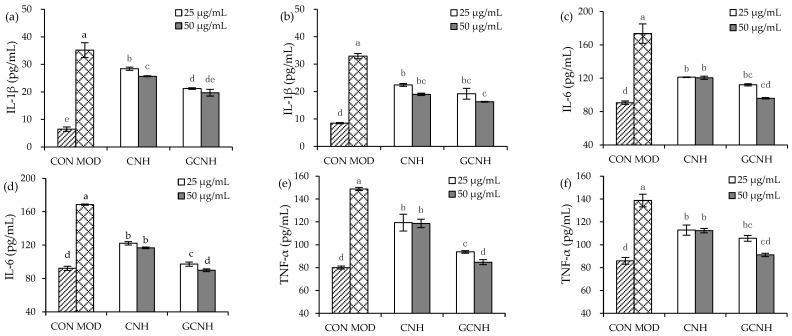
Secretion levels of three pro-inflammatory mediators IL-1β (**a**,**b**), IL-6 (**c**,**d**), and TNF-α (**e**,**f**) in the IEC-6 cells incubated with casein hydrolysates (CNH) and oligochitosan-glycated CNH (GCNH) at doses of 25–50 μg/mL for 12 (**a**,**c**,**e**) and 24 h (**b**,**d**,**f**), respectively, followed by LPS treatment (10 μg/mL) of 24 h. The abbreviations “CON” and “MOD” stand for the control and LPS-stimulated cells, respectively, while different lowercase letters above the columns indicate that the mean values differ significantly (*p* < 0.05).

Secretion levels of three inflammatory mediators (IL-1β, IL-6, and TNF-α) were detected in the supernatants collected from the treated cells (Figure 3 and Figure 4), to clarify whether CNH and GCNH had different effects on the cells in cytokine secretion. First, the two hydrolysates in IEC-6 cells did not promote IL-6 secretion (Figure 3), indicating they did not cause inflammation in the cells. However, the two hydrolysates in the LPS-induced cells showed a capacity to inhibit the secretion of the three inflammatory mediators (Figure 4). Specifically, the LPS-induced cells showed IL-1β, IL-6, and TNF-α secretion of 32.2–33.0, 168.5–173.5, and 138.8–148.8 pg/mL, while the CNH-treated cells (12 h) after LPS stimulation showed reduced IL-1β, IL-6, and TNF-α secretion of 25.7–28.4, 120.6–121.2, and 118.7–119.4 pg/mL, respectively. In addition, the GCNH-treated cells (12 h) after LPS stimulation had much reduced IL-1β, IL-6, and TNF-α secretion of 19.3–21.7, 96.0–112.2, and 84.8–93.8 pg/mL, respectively. Using a 24 h of cell treatment time with the two hydrolysates, the CNH-treated cells after LPS stimulation showed further reduction in IL-1β, IL-6, and TNF-α secretion (19.0–22.4, 116.8–122.2, and 112.4–112.8 pg/mL), while the GCNH-treated cells after LPS stimulation had the lowest secretion in IL-1β, IL-6, and TNF-α (16.3–23.2, 89.8–97.2, and 91.2–105.7 pg/mL). These data proved consistently that the two hydrolysates had an anti-inflammatory effect on the LPS-induced cells by inhibiting the secretion of the three inflammatory mediators, and more interestingly, GCNH were more capable of reducing inflammatory cytokines secretion than CNH. It was thus suggested that the employed oligochitosan glycation of casein led to enhanced anti-inflammatory potential for the obtained GCNH in the LPS-induced cells.

### 3.4. Effect of Two Hydrolysates on the Secretion of Anti-Inflammatory Mediators

**Figure 5:**
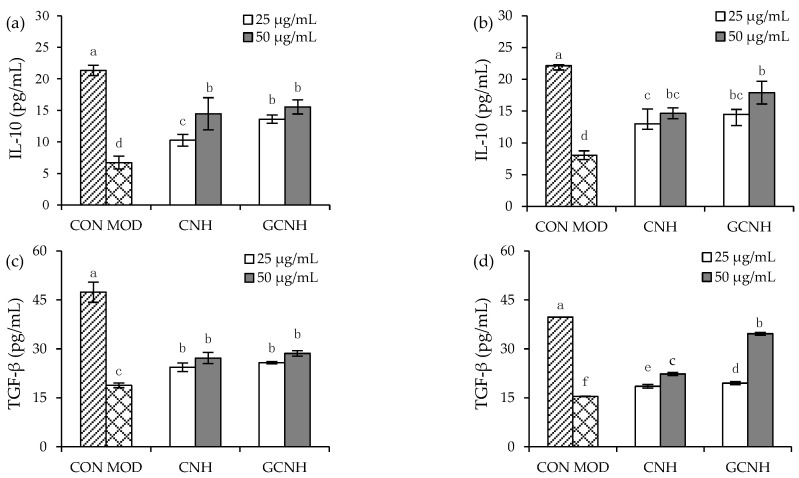
Secretion levels of two anti-inflammatory mediators IL-10 (**a**,**b**) and TGF-β (**c**,**d**) in the IEC-6 cells incubated with the casein hydrolysates (CNH) and oligochitosan-glycated CNH (GCNH) at doses of 25–50 μg/mL for 12 (**a**,**c**) and 24 h (**b**,**d**), respectively, followed by LPS treatment (10 μg/mL) of 24 h. The abbreviations “CON” and “MOD” stand for the control and LPS-stimulated cells, respectively, while different lowercase letters above the columns indicate that the mean values differ significantly (*p* < 0.05).

Compared with the secretion of the two anti-inflammatory mediators IL-10 and TGF-β in the control cells, the present results showed that LPS caused lower secretion in IL-10 and TGF-β in the LPS-induced cells (Figure 5), because IL-10 (or TGF-β) level was decreased from 21.3–22.2 to 6.7–8.1 pg/mL (or from 39.7–47.4 to 15.4–18.8 pg/mL). On the contrary, CNH and GCNH consistently promoted the secretion of IL-10 and TGF-β in the LPS-induced cells. In detail, when the cells were treated by CNH for 12 h before the LPS exposure, they were measured with increased IL-10 (10.27–14.46 pg/mL) and TGF-β (24.35–27.19 pg/mL) secretion. Meanwhile, if the cells were pre-treated with GCNH for 12 h before the LPS exposure, they were measured to have distinctly increased IL-10 and TGF-β secretion of 13.6–15.6 and 25.8–28.6 pg/mL, respectively. In addition, the CNH-treated cells (24 h) after the LPS exposure had much secretion increases in IL-10 and TGF-β (13.0–14.7 and 18.5–22.3 pg/mL), while the GCNH-treated cells (24 h) after the LPS exposure had the highest secretion increases in IL-10 and TGF-β (14.5–17.9 and 19.5–34.6 pg/mL). The data changes demonstrated that the two hydrolysates had an anti-inflammatory effect on the LPS-induced cells in all cases because they consistently enhanced the secretion of IL-10 and TGF-β. Further data comparison also suggested that GCNH were more efficient than CNH at promoting the secretion of IL-10 and TGF-β. This fact evidenced again that the performed oligochitosan glycation of casein could enhance the anti-inflammatory activity of GCNH, via endowing GCNH with higher potential to promote the secretion of anti-inflammatory mediators IL-10 and TGF-β.

### 3.5. Expression Changes of the Signaling Pathway-Related Proteins

**Figure 6:**
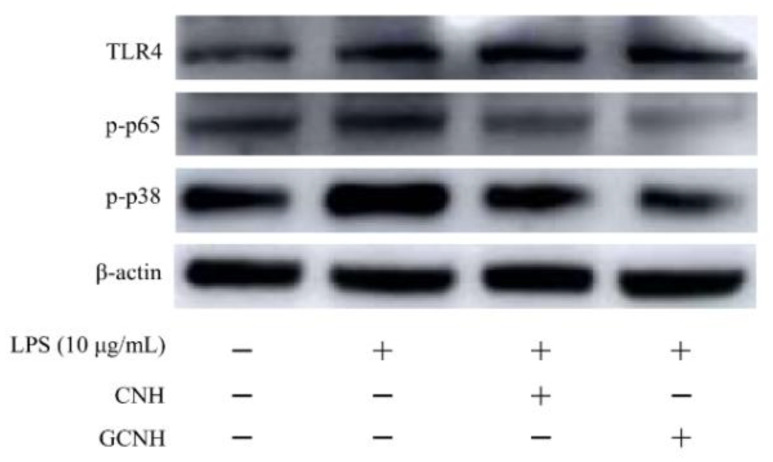
Expression of three proteins TLR4, p-p65, p-p38 in the IEC-6 cells incubated with or without casein hydrolysates (CNH) and oligochitosan-glycated CNH (GCNH) at doses of 25–50 μg/mL for 24 h or LPS treatment (10 μg/mL) of 24 h.

**Figure 7:**
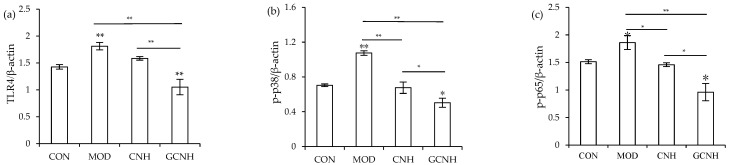
The calculated ratios of TLR4/β-actin (**a**), p-p38/β-actin (**b**), and p-p65/β-actin (**c**) in the IEC-6 cells incubated with or without casein hydrolysates (CNH) and oligochitosan-glycated CNH (GCNH) at doses of 25–50 μg/mL for 24 h or LPS treatment (10 μg/mL) of 24 h. The abbreviations “CON” and “MOD” stand for the control and LPS-stimulated cells, respectively, while the labeled asterisks indicate a significant difference (* *p* < 0.05 and ** *p* < 0.01).

To further clarify whether CNH and GCNH had anti-inflammatory activities to the LPS-induced cells, the expression levels of three proteins TLR4, p-p65 and p-p38 from the TLR4-NF-κB/MAPK signaling pathway, which is regarded to play a critical role in cellular inflammation, were thus detected. The immunoblotting results (Figure 6 and Figure 7) showed that when the ratios of TLR4/β-actin, p-p65/β-actin, and p-p38/β-actin were used as three evaluation indicators, the two hydrolysates were capable of down-regulating the expression levels of TLR4, p-p65 and p-p38. Compared with the control cells, the LPS-induced cells had up-regulated expression for TLR4, p-p65 and p-p38 by 1.81-, 1.86-, and 1.07-folds, respectively. Meanwhile, the CNH-treated cells after LPS stimulation showed a reduced expression for TLR4, p-p65, and p-p38 (1.59-, 1.46-, and 0.68-folds), whilst the GCNH-treated cells after the LPS stimulation showed the lowest expression for TLR4, p-p65 and p-p38 (1.05-, 0.96-, and 0.50-folds). The p-p65 and p-p38 are key proteins of cellular inflammation in the NF-κB and MAPK signaling pathways, respectively. Thus, the western-blotting results revealed that the two hydrolysates could inhibit the LPS-induced pathway activation by down-regulating the critical proteins TLR4, p-p65, and p-p38, and thus possessed anti-inflammatory activities to the induced cells. In addition, GCNH led to a higher reduction in the expression of the three proteins in the induced cells, implying that GCNH might have higher anti-inflammatory activity than CNH. In other words, the performed oligochitosan glycation showed the ability to increase the anti-inflammatory potential of GCNH.

## 4. Discussion

Food proteins are essential for body growth and health maintenance, as they provide both nutrition and bioactivities in the body. Protein glycation could alter the properties of the protein targets efficiently. In referring to the protein glycation of the Maillard-type, it was evidenced that this glycation could improve solubility, rheology, emulsification, thermal stability, foaming, and gelation of the treated proteins [36,37], or enhance the anti-bacterial, anti-oxidant, ACE-inhibitory, and anti-inflammatory activities of the peptides [38]. Unfortunately, a previous study also found that lactose glycation of casein led to lower immune potential for the casein hydrolysates [4]. TGase can also catalyze the reaction between proteins and amino sugars, causing another reaction type of protein glycation (i.e., protein glycation of TGase-type). The results indicated that TGase catalyzed the glycation of ferritin by oligochitosan, while the grafted amount of amino sugar was 5.6–8.3 g/kg protein [39]. Moreover, the glycated ferritin obtained higher thermal stability because its maximum peak temperature was increased from 73.12 °C to 78.16 °C [39]. In addition, when TGase was used to catalyze the glycation of casein by the degraded chitosan, the surface hydrophobicity, in vitro digestibility, water-binding, solubility, and rheological properties of the modified casein were improved [1,40]. Therefore, whether the TGase-type protein glycation induced an alteration in protein bioactivities aroused extensive attention in the past years. It was observed that the chitosan-glycation of hemoglobin by TGase resulted in respective 11.06% and 30.49% increases in anti-oxidant activity and iron bioavailability [41], while the glucosamine-glycation of bovine β-lactoglobulin by TGase led to a weakened allergic reactivity including a reduced IgG/IgE binding capacity [4]. More importantly, the previous results from our groups also indicated that oligochitosan-glycation of casein by TGase brought about higher barrier protection for the casein hydrolysates in IEC-6 cells [6,33]. All results thus supported that GCNH had higher bioactivity than CNH with which to alleviate the LPS-induced cell inflammation in the target cells because GCNH were yielded from the enzymatic hydrolysis of the oligochitosan-glycated casein, while the oligochitosan molecules were evidenced to be conjugated into the Gln residues of the glycated casein hydrolysates [33].

It was fully proved that protein hydrolysates had various bioactivities, and thus could perform critical anti-oxidant, anti-microbial, anti-hypertensive, and anti-diabetic effects [42]. For example, when camel milk protein was hydrolyzed under the catalysis of three proteases like alkaline protease, α-chymosin and papain, the yielded protein hydrolysates showed anti-oxidant activity to scavenge DPPH/ABTS radicals and to reduce iron ions [43]. The isolated components from camel protein hydrolysates by protease K were reported to have ACE inhibitory activity [44], while the albumin-derived peptide KLPGF was confirmed to have anti-diabetic potential through inhibiting α-glucosidase and α-amylase [45]. In addition, the hydrophobic peptides separated from soybean protein hydrolysates were found to have an anti-cancer effect [46]. It has been well-established that protein hydrolysates can regulate the immune response in cells and tissues. It was observed that the hydrolysates from shark proteins were able to enhance the phagocytosis activity of macrophages, or to increase the levels of IgA as well as IFN-γ, TNF-α, IL-10, IL-4 and IL-6 in small intestine tissues, confirming the immuno-modulatory effect of the hydrolysates [47]. Furthermore, the pea protein hydrolysates could enhance the phagocytosis activity of mouse macrophages, or stimulate intestinal cells to produce IL-6 [48]. Inflammation is a transient phenomenon of the immune response, and the anti-inflammatory effects of protein hydrolysates have been assessed sufficiently. For example, the sturgeon protein peptides showed anti-inflammatory effect on the LPS-induced macrophages by reducing the release of NO, IL-6, and IL-1β, or inhibiting the activation of MAPK signaling pathway [20,49]. It was found that casein glycopeptide hydrolysates could neutralize LPS by directly binding to LPS or inhibiting the binding of LPS to the TLR4/MD2 complex, causing an inactivation of TLR4/MyD88/NF-κB and less production of TNF-α and IL-1β [50]. Casein glycopeptide hydrolysates in the LPS-stimulated RAW264.7 cells were thus regarded to possess anti-inflammatory function by modulating the TLR4-mediated inflammatory responses [50]. Another previous study also confirmed that whey protein hydrolysates by pepsin, trypsin, chymotrypsin and peptidase had anti-inflammatory effect on the LPS-induced inflammation in respiratory epithelial cells, through inhibiting the secretion of pro-inflammatory mediator IL-8 [51]. In this study, the target GCNH also belonged to protein hydrolysates, and reasonably had similar anti-inflammatory function in the LPS-stimulated IEC-6 cells. However, due to the oligochitosan conjugation into casein molecules, GCNH reasonably contained some glycated peptides. Shared result consistence with these reported studies [50,52,53], this study also observed that GCNH had ant-inflammatory function in the LPS-stimulated IEC-6 cells, and also showed higher potential than CNH.

Inflammatory response can induce the formation of cytokines and inflammatory mediators [54]. Several bioactive components in dietary foods like proteins, polysaccharides, and polyphenols have anti-inflammatory potentials, via regulating the secretion of inflammatory (IL-1β, IL-6, and TNF-α) and anti-inflammatory cytokines (IL-10 and TGF-β). Thus, the secretion levels of these cytokines can be directly used to reflect the anti-inflammatory potential of assessed targets. The previous results showed that the secretion levels of IL-1β, IL-6, and TNF-α were enhanced, when the RAW 264.7 macrophages were exposed to LPS [20]. Using these indicators, the mung protein hydrolysate [19], casein glycopeptide hydrolysate [50], and strawberry polysaccharides [22] were regarded to have anti-inflammatory function because they were able to reduce the levels of IL-1β, IL-6 and TNF-α but increase the levels of IL-10. Two previous studies have also used the secretion levels of inflammatory cytokines IL-1β, IL-6, and TNF-α of macrophages as indicators to identify the anti-inflammatory effects of polyphenols from sea buckthorn [21] and citrus peel [55]. It is worth noting that LPS-induced cellular inflammation can also be observed in IEC-6 cells. For example, bovine lactoferrin had anti-inflammatory function in the stimulated IEC-6 cells by reducing the mRNA levels or protein expression of IL-1β, IL-6, and TNF-α [24]. In addition, when IEC-6 cells were induced by LPS, the levels of TNF-α and IL-1β were 330 and 279 pg/mL, respectively; however, when β-carotene (50 μmol/L) was used to treat the cells, the levels of TNF-α and IL-1β were reduced by 45% and 55%, respectively [23]. It is now known that the effect of cytokines on inflammation in cells is closely related to the expression of extracellular signaling pathway proteins. For the NF-κB signaling pathway, in response to LPS stimulation, phosphorylation of IκBα causes the translocation of the free p65 subunit into the nucleus, triggering a series of cytokine secretion [56]. P38 and JNK proteins of MAPK signaling pathways are important in cellular inflammation [30]. These proteins are key elements to the transmission of signals from the cell surface to the nucleus, and an inhibition of these proteins can effectively prevent the occurrence of cell inflammation. Thus, it was observed that p-p65 and p-p38 protein expressions were significantly inhibited when the two polysaccharides from *Pleurotus eryngii* were used to treat LPS-induced macrophages [57]. When LPS-induced IEC-6 cells were treated with β-carotene, the relative phosphorylation level of p38 protein was decreased from 0.7 to 0.3 [23]. In the LPS-induced macrophages, casein glycopeptide hydrolysates also showed anti-inflammatory effect by inhibiting the expression of TLR4 and p-p65 proteins [50]. In consistent with these mentioned studies [23,50,57], this study also found that the two hydrolysates possessed anti-inflammatory activities to the LPS-stimulated cells via mediating cytokine secretion, together with a regulation on protein expression in the target signaling pathway.

## 5. Conclusions

The results from this study indicated that the enzymatic oligochitosan-glycation of casein incorporated oligochitosan molecules into the protein target, and thus brought about enhanced anti-inflammatory activities for the obtained glycated casein hydrolysates to the LPS-induced IEC-6 cells. Overall, the glycated casein hydrolysates had no cytotoxic effect on the cells but could alleviate the LPS-induced inflammation by increasing the secretion of two anti-inflammatory mediators, decreasing the secretion of three pro-inflammatory mediators, and down-regulating the expression of three inflammation-related proteins. Moreover, the glycated casein hydrolysates were more effective than the unglycated casein hydrolysates at producing these anti-inflammatory functions. Thus, the protein glycation using oligochitosan and transglutaminase might be a potential way to endow protein hydrolysates with higher anti-inflammatory potential in the intestine to antagonize the LPS-induced inflammation in intestinal epithelial cells, demonstrating this modification is applicable to generate protein ingredients with higher healthcare function in the body.

## Data Availability

All data are contained within the article.
